# Melatonin as a Chronobiotic and Cytoprotective Agent in Parkinson’s Disease

**DOI:** 10.3389/fphar.2021.650597

**Published:** 2021-04-15

**Authors:** Santiago Pérez-Lloret, Daniel P. Cardinali

**Affiliations:** ^1^ Universidad Abierta Interamericana–Centro de Altos Estudios en Ciencias Humanas y de La Salud, Consejo Nacional de Investigaciones Científicas y Técnicas, UAI-CAECIHS. CONICET, Buenos Aires, Argentina; ^2^ Faculty of Medical Sciences, Pontificia Universidad Católica Argentina, Buenos Aires, Argentina

**Keywords:** aging, glymphatic system, melatonin, neurodegeneration, oxidative stress, parkinson’s disease, sleep

## Abstract

This article discusses the role that melatonin may have in the prevention and treatment of Parkinson’s disease (PD). In parkinsonian patients circulating melatonin levels are consistently disrupted and the potential therapeutic value of melatonin on sleep disorders in PD was examined in a limited number of clinical studies using 2–5 mg/day melatonin at bedtime. The low levels of melatonin MT1 and MT2 receptor density in substantia nigra and amygdala found in PD patients supported the hypothesis that the altered sleep/wake cycle seen in PD could be due to a disrupted melatonergic system. Motor symptomatology is seen in PD patients when about 75% of the dopaminergic cells in the substantia nigra pars compacta region degenerate. Nevertheless, symptoms like rapid eye movement (REM) sleep behavior disorder (RBD), hyposmia or depression may precede the onset of motor symptoms in PD for years and are index of worse prognosis. Indeed, RBD patients may evolve to an α-synucleinopathy within 10 years of RBD onset. Daily bedtime administration of 3–12 mg of melatonin has been demonstrated effective in RDB treatment and may halt neurodegeneration to PD. In studies on animal models of PD melatonin was effective to curtail symptomatology in doses that allometrically projected to humans were in the 40–100 mg/day range, rarely employed clinically. Therefore, double-blind, placebo-controlled clinical studies are urgently needed in this respect.

## Introduction

With a prevalence of 1–4% in people >60 years of age, Parkinson’s disease (PD) is the second most common neurodegenerative disorder (Tysnes and Storstein, 2017). Both genetic and environmental factors contribute to the evolution and progression of PD as a complex multifactorial disease. Indeed, several studies have pointed out to the involvement of genetic factors in the pathogenesis of PD, but much less is known about the contribution of environmental and lifestyle factors like e.g. circadian and sleep changes. Current PD diagnosis relies on the identification of motor clinical signs and symptoms, including bradykinesia, rigidity and rest tremor. In addition, patients with PD develop a wide range of non-motor manifestations including sleep and mood disturbances, cognitive impairment and dementia, and autonomic nervous system dysfunction many times in advance to motor dysfunction (Poewe et al., 2008). Since a loss of 50–80% of dopaminergic neurons in the substantia nigra pars compacta is needed for the first motor signs occur (Cheng et al., 2010), neuroprotection strategies at this stage tends to be unsuccessful. because brain injury has been ongoing for years.

A multifactorial etiology of PD as a neurodegenerative disorder is advocated. The loss of dopaminergic neurons has been attributed to a low-degree inflammation and oxidative damage (Abramov et al., 2020; Singh and Devasahayam, 2020). As main mediators of inflammatory responses, the microglia becomes activated and release reactive oxygen species and proinflammatory cytokines in PD (Kam et al., 2020). In addition, a dysfunctional blood brain barrier is involved in the progression of the disease and a faulty metabolism and elimination of putative PD toxins by the glymphatic system occur (Sundaram et al., 2019). This is associated with infiltration of peripheral immune cells resulting in enhanced inflammation. Excessive accumulation of iron in the substantia nigra is another factor reported to have a pathogenic role in PD as it promotes enhanced generation of reactive oxygen species (Mochizuki et al., 2020). Hence, the effectiveness of administering antioxidants for neurodegeneration prevention has been proposed but albeit questioned (Davies et al., 2017).

Melatonin is present in all aerobic phyla and may have therapeutic significance both as a cytoprotective and chronobiotic properties (Cardinali, 2019b). The pineal gland accounts for more than 95% of circulating melatonin levels and the decrease of these levels is typical of advancing age in humans (Claustrat and Leston, 2015). In this article we will review the clinical use of melatonin in PD, with the discussion of animal studies being restricted to their relevance for the melatonin doses used in humans. Melatonin’s cytoprotective effect and clinical usefulness in neurodegenerative disorders are discussed elsewhere (Cardinali, 2019b).

## Alterations in Biological Rhythms in Parkinson’s disease and Its Relevance for the Neurodegenerative Process

There is abundant evidence pointing out to the existence of disturbances in biological rhythms in PD. In one study, bilateral nigrostriatal lesions with 6-hydroxydopamine (6-OHDA) in rats disrupted heart rate, temperature and activity rhythms (Ben and Bruguerolle, 2000). Lesioned animals showed significantly decreased mean daily values and a phase advance of circadian rhythms. Decreased amplitude was also observed in heart rate rhythms. Abolition of heart rhythms was also observed in another study in 6-OHDA-lesioned rats (Henderson et al., 2010). However, alterations in biological rhythms were not observed after 1-methyl-4-phenyl-1,2,3,6-tetrahydropyridine (MPTP) lesions in C57bl/6 mice maintained in light: dark (L:D) 12:12 (Leng et al., 2004)) or in non-human primates (Barcia et al., 2004) (REF). Notwithstanding, in the latter, locomotor activity and hormonal rhythms alterations in become evident when animals were challenged by exposure to constant light (Fifel et al., 2014). Administration to rats placed in L:D 12:12 of rotenone, a pesticide with toxic effects in dopaminergic neurons, produced a reduction in the mean value and amplitude of the circadian locomotor activity and body temperature rhythms (Lax et al., 2012). Lower interdaily phase stability, and higher rhythm fragmentation were also observed in rats.

The exploration of biological rhythms in transgenic models of parkinsonism have yielded interesting results. A study in a transgenic mouse PD model overexpressing alpha-synuclein, found deposition of this protein in the suprachiasmatic nuclei (SCN), which bears the master biological clock (Kudo et al., 2011). Results in these animals suggested a weakened circadian system time, which may thus be a core feature of PD.

Results in experimental parkinsonism also show altered expression of the clock genes, Indeed, reductions in PER2, which is one of the genes that control rhythms at a cellular level, levels were observed in the dorsal striatum of 6-OHDA lesioned rats (Hood et al., 2010). Circadian expression of clock genes in the SCN were also abolished after rotenone administration to rats (Lax et al., 2012).

Antiparkinsonian medications used to treat PD may impact on the biological rhythms on their own. In 6OHDA-lesioned, treatment with l-dihydroxyphenylalanine (l-DOPA) produced an increase in mean daily values of temperature and heart rate (Simon et al., 2000). While the temperature rhythm’s amplitude was increased, heart rate rhythm’s amplitude was reduced. Interestingly, these effects were in opposition to those imposed by 6-OHDA lesions (Ben and Bruguerolle, 2000). Thus, l-DOPA corrected to some extent the alterations of temperature and heart rate rhythms. Further benefits may be offered by circadian infusions of l-DOPA, if they are capable to mimic the normal dopamine rhythm (Boulamery et al., 2010). Alterations in biological rhythms have been also observed in the transgenic mouse MitoPark model of PD (Kudo et al., 2011).

The hypothesis that biological rhythms could, in turn, affect the development of experimental parkinsonism was tested in a study in which enucleation of the lateral hypothalamus was performed in the 6-OHDA-lesioned rats kept in L:D 12:12 (Fifel and Cooper, 2014). Removal of the lateral hypothalamus disrupts the retino-hypothalamic tract and leads to altered biological rhythms. Compared to 6-OHDA lesions alone, removal of the lateral hypothalamus ipsilateral to the side of the 6-OHDA lesion, was associated with impairments of horizontal movement, limb retraction, ambulation and spontaneous or l-DOPA induced turning (Willis et al., 2008).

Disturbances of biological rhythms have also been found in PD patients. For example, lower expression of BMAL1 has been observed in patients (Cai et al., 2010; Ding et al., 2011; Breen et al., 2014). Furthermore, polymorphism in ARNTL and PER1 genes were observed more frequently in a sample of 1253 Chinese PD cases as compared to 1342 healthy controls, thus reinforcing the idea that circadian alterations might contribute to the neurodegenerative process in PD. One study reported alterations in the body temperature rhythm, which were correlated to the self-reported severity of Rapid Eye Movement (REM) Sleep Behavior Disorder (RBD) symptoms (Zhong et al., 2013). Alterations in cortisol circadian rhythm have also been documented in PD, with elevated levels of this hormone during nighttime and early morning (Hartmann et al., 1997). Disturbances in the blood pressure rhythm, with nighttime hypertension, have also been observed (Ahsan Ejaz et al., 2006; Berganzo et al., 2013).

## Sleep Disorders in Parkinson’s disease

Sleep disorders affect PD patients frequently and impact negatively on quality of life (Gallagher et al., 2010; Avidan et al., 2013). Insufficient sleep interferes with routine activities and can also aggravate motor symptoms in PD. Some of the most frequent disorders are RBD, insomnia, restless legs syndrome (RLS) and periodic limb movement disorder, circadian rhythm sleep disorders (CRSD), nocturia, sleep disordered breathing (SBD) and excessive daytime sleepiness (EDS). In the following paragraphs we will review the essential aspects of each condition.

RBD is a REM parasomnia characterized by movements occurring during REM sleep, in the context of dreaming (i.e. patients “play along” their dreams). Somatic muscle atonia occurs normally during REM sleep, which is lost in RBD. RBD prevalence in PD ranges between 39 and 46% (Sixel-Döring et al., 2011; Neikrug et al., 2013). In some instances, RBD may precede the diagnosis of PD, but PD patients may also develop this condition after receiving the clinical diagnosis. For establishing the diagnosis of RBD, a history or visualization of dream enactment along with polysomnographic evidence of REM sleep without atonia is required (American Academy of Sleep Medicine, 2015). Dream enactment can be assessed by means of screening questionnaires, but they demonstrate low specificity.

Insomnia can be defined as inadequate sleep quality reported subjectively. It constitutes considered a symptom resulting from different pathological processes. In PD, the most commonly reported complaint is sleep fragmentation (Gómez-Esteban et al., 2006). Poor sleep in PD has been reported in between 20 and 80% of patients (Gjerstad et al., 2007; Porter et al., 2008). The assessment of insomnia is achieved using questionnaires. Parkinson’s disease Sleep Scale (PDSS), Scales for Outcomes in Parkinson’s disease Sleep (SCOPA-Sleep), and the Pittsburgh Sleep Quality Index (PSQI) may be used for the subjective assessment of sleep in PD (Högl et al., 2010). The PDSS and SCOPA-Sleep scales were conceived specifically for the assessment of PD patients (Chaudhuri et al., 2002; Marinus et al., 2003). The SCOPA-Sleep is shorter and thus easier to use. The PSQI is commonly used to assess sleep quality in PD or other groups of patients (Buysse et al., 1989). Polysomnography may be indicated with another primary sleep disorder, such as sleep apnea, is suspected.

RLS is a sleep-related movement disorder that may affect patients with PD, with a prevalence of 3–21.3% (Loo and Tan, 2008; Azmin et al., 2013; Rana et al., 2013). Some studies suggest RLS may be a risk factor to develop PD. However, the opposite has been observed (i.e., PD being a risk factor for RLS) (Calzetti et al., 2014; Wong et al., 2014) The diagnosis of RLS requires the presence of an urge to move the legs, which may be accompanied by an uncomfortable sensation (American Academy of Sleep Medicine, 2015). This sensation occurs with rest/inactivity, most commonly at night or in the evening, and is at least partially relieved by movement. According to the ICSD III criteria, significant distress or result in sleep impairment should also result from symptoms. An immobilization test has been developed as an objective marker of RLS (De Cock et al., 2012). Patients are instructed to lay still for 1 h at night. They are requested to report the severity of leg discomfort on a scale of 0 (no discomfort) to 100 (extreme discomfort) every 10 min. Notwithstanding, its utility for detecting alternative diagnoses of RLS in PD has not been evaluated.

PLMD refers to the presence of periodic limb movements during sleep (PLMS), which lead to significant impairment of sleep and/or functioning (American Academy of Sleep Medicine, 2015). The diagnosis of PLMD requires >15 PLMS per hour. While PLMS are frequently seen in patients with RLS; they can also be seen in isolation in the elderly population (Ancoli-Israel et al., 1991).

CRSD is characterized by disturbances of the 24-hs sleep-wake rhythm. As mentioned earlier, there is a considerable alteration of the biological clock in PD. The impact of CRSD in PD has only been recently appreciated, and further research is warranted.

Nocturia is reported in 60–80% of PD patients (Winge et al., 2006). Risk factors for nocturia include male sex, older age, older age of PD onset, and increased PD duration and severity (Rana et al., 2014). The pathogenesis of this condition is multifactorial. Detrusor muscle hyperactivity and polyuria may be related to nocturia (Xue et al., 2014). Dopaminergic medications, sleep apnea, and other factors or conductions may aggravate the syndrome (Picillo et al., 2014). Assessment of nocturia in PD patients can be performed by questionnaires, such as The Movement Disorders Society-Unified Parkinson’s disease Rating Scale (MDS-UPDRS), the Parkinson’s disease sleep scale version-2 and SCOPA-sleep (Marinus et al., 2003; Trenkwalder et al., 2011).

SDB affects 15–76% of PD patients (Chahine et al., 2017). Obstructive and restrictive pulmonary pathology as well as an abnormal response to hypercapnia, are the most frequently described patterns of SDB in PD (Sabaté et al., 1996; Monteiro et al., 2012). PD patients may be prone to develop SDB after developing pulmonary dysfunction. Polysomnography is required for the diagnosis of SDB.

Finally, EDS may affect 20–60% of PD patients (Chahine et al., 2017). EDS may precede PD diagnosis or may be observed during the course of treatment with dopaminergic agents (Buskova et al., 2011) EDS has a considerable impact in daily activities, quality of life, and caregiver burden (Ozdilek and Gunal, 2012). The Epworth sleepiness scale (ESS) is the measure of choice for the assessment of EDS in PD (Hagell and Broman, 2007). Objective assessment of EDS may be performed by means of the multiple sleep latency test (MSLT) and maintenance of wakefulness test (MWT). These tests evaluates the ability of the subject to maintain wakefulness or to fall asleep, respectively (American Academy of Sleep Medicine, 2015).

## Basic Biology of Melatonin Relevant to Parkinson’s disease

“Chronobiotics” are drugs that synchronize or increase the amplitude of circadian rhythms, melatonin being its natural prototype (Dawson and Armstrong, 1996; Cardinali et al., 2008). The essential role of melatonin as a chronobiotic is defined by its prominent light/dark rhythm in circulation. Melatonin inhibits the wakefulness-facilitating activity of the SCN in the late afternoon, triggering sleep (Lavie, 1997; Lewy et al., 2006). As the secretion of pineal melatonin is proportional to the length of the night, it provides the neuroendocrine apparatus with fundamental information on the time of year regulate ng seasonality (Pandi-Perumal et al., 2008).

Although more than 90% of circulating melatonin derives from the pineal gland (Claustrat and Leston, 2015) there is ample evidence that it is synthesized locally in most cells, tissues, and organs (Acuña-Castroviejo et al., 2014). Indeed, the idea is held that that melatonin is produced in all animal cells that have mitochondria (Tan and Reiter, 2019) and that a mitochondrial protective function is critical for cytoprotection (Hardeland et al., 2011).

Melatonergic receptors (MT1 and MT2) are responsible for the chronobiotic function of melatonin. Both receptors are members of the superfamily of membrane receptors associated with G proteins (G-protein-coupled receptors, GPCR) (Dubocovich et al., 2010). A third member of the melatonin receptor family, GPR50, displays high sequence homology with MT1 and MT2 but rather than binding melatonin or any other known ligand, it forms homo and heteromers with MT1 and MT2 as well as with other GPCRs (Cecon et al., 2018).

MT1 and MT2 receptors have been identified in SCN and in several CNS areas, including the cerebral and cerebellar cortex and the midbrain (Ng et al., 2017). In the midbrain, MT1 and MT2 receptors are related to modulation of the nigrostriatal and mesocorticolimbic dopaminergic pathways, possibly related to the pathophysiology of PD. Functional melatonin receptors have been localized in substantia nigra, nucleus accumbens, ventral tegmental areas and caudate-putamen (Uz et al., 2005). The substantia nigra of PD patients exhibited a decreased expression of MT1 and MT2 receptors (Adi et al., 2010a).

Due to its amphiphilic properties melatonin crosses readily cell membranes. Calmodulin and tubulin are among the cytoplasmic proteins that interacts with melatonin (Jiménez-Rubio et al., 2012). Melatonin also enters the cell nucleus and interacts indirectly with orphan RZR/ROR superfamily of receptors (Hardeland et al., 2011) through the activation of sirtuin-1, which affects the circadian accessory oscillator RORα 1 (Hardeland, 2018b).

The cytoprotective activity of melatonin cannot be explained by interaction with MT1 and MT2 receptors only. As mentioned, the amounts of intracellular melatonin greatly exceeds those found in circulation (Acuña-Castroviejo et al., 2014). Intramitochondrially synthesized melatonin is retained within the organelle and the melatonin doses capable to modify the intracellular melatonin concentration are much higher than those used clinically as a chronobiotic (Venegas et al., 2013).

Although in cell cultures, some physiologically relevant effects of melatonin are revealed at doses in the range of 10^−8^–10^–9^ M (Zemková and Vaněček, 1997) for most studies on cytoprotective effects in animals pharmacological doses that clearly exceed receptor saturation were employed (Cardinali, 2019a).

This review focuses on the effects of melatonin on neurodegeneration in animal models of PD in relation to the possible human doses to be employed. For recent reviews related to the activity of melatonin to reverse altered signaling mechanisms in neurodegeneration, including impaired autophagic integrity, proteostasis dysfunction and abnormalities in Notch, Wnt/ß and insulin signaling pathways see (Shukla et al., 2019; Tamtaji et al., 2020; Tan et al., 2020).

The melatonin antioxidant and scavenging effects on free radicals occurring in both the cytoplasm and the cell nucleus, are largely independent of receptors. Besides being a free radical scavenger itself, there is an oxidation-drive conversion of melatonin to compounds with higher antioxidant activit. In addition, melatonin displays a strong indirect antioxidant effect by stimulating the synthesis of antioxidant enzymes and by inhibiting that of pro-oxidant enzymes. In protecting against oxidative damage melatonin is more effective that vitamin C and E (Reiter et al., 2017). Moreover, melatonin facilitates the effects of other antioxidants, like Trolox or vitamin C. A stabilizing activity of melatonin on mitochondrial membrane explain several cytoprotective and antiapoptotic effects exerted by melatonin under ischemic conditions (not related to free radicals) (Tan and Reiter, 2019).

Immunomodulation by melatonin includes pro-inflammatory and anti-inflammatory effects (Carrillo-Vico et al., 2013; Hardeland, 2018a). Anti-inflammatory actions of melatonin have been demonstrated in low-grade inflammation, like neurodegenerative processes or aging as well as in high-grade inflammation such as sepsis, ischemia/reperfusion or brain injury.

Melatonin anti-inflammatory properties are related to the inhibition of nuclear factor κB (NF κB) binding to DNA, and of cyclooxygenase (Cox) (Cardinali et al., 1980) (mainly Cox-2) (Deng et al., 2006)), as well as to down-regulation of inducible nitric oxide synthase receptors (Hardeland et al., 2011). Other signaling pathways include up-regulation of nuclear factor erythroid 2-related factor 2 and of toll-like receptor-4 activation and high-mobility group box-1 signaling receptors, and prevention of NLRP3 inflammasome activation by melatonin (Hardeland et al., 2011). Collectively, these effects of melatonin result in increased production of anti-inflammatory cytokines and reduced levels of pro-inflammatory cytokines (Carrillo-Vico et al., 2013; Hardeland, 2018a).

In PD patients, γ-aminobutyric acid (GABA)-ergic dysregulation in basal ganglia has been documented by means of magnetic resonance spectroscopy (Huang et al., 2019). Since striatal dopaminergic axons release GABA, dopaminergic neurodegeneration may lead to a GABA hypofunction in basal ganglia circuits (O’Gorman Tuura et al., 2018). The trigger of neurodegenerative processes could be followed by a loss of the inhibitory tone of GABA and accumulation of abnormal levels of intracellular calcium. In support of this view, the GABA agonist baclofen has been shown to alleviate motor symptoms and protect dopamine cell bodies in a PD murine model (Lozovaya et al., 2018).

The GABAergic system is involved in melatonin-mediated neuroprotection. In fact, melatonin exerts anti-excitatory and sedative effects (Golombek et al., 1996; Caumo et al., 2009) and there is information that melatonin protects neurons from ß-amyloid peptide toxicity through the activation of GABAergic receptors (Louzada et al., 2004). An allosteric modulation of GABA_A_ receptors by melatonin was indicated by the efficacy of the benzodiazepine antagonist flumazenil, and the lack of activity of the melatonin receptor antagonist luzindole, to modify upregulation of GABA activity by melatonin (Cheng et al., 2012).

The anti-excitotoxic activity of melatonin should be also considered. For example, melatonin administration protects CA1 neurons in the hippocampus from transient ischemia (Cho et al., 1997) or from death due to high doses of glucocorticoids (Furio et al., 2014). Kainate, an agonist of the ionotropic glutamate receptor, induces neuronal death, an effect also prevented by melatonin (Giusti et al., 1996). As revealed by the lack of effects of specific blockers, melatonin receptors do not participate in the anti-excitotoxic activity of melatonin (Escames et al., 2004).

Finally, the recognized activity of melatonin in reversing the effects of an increased insulin resistance (IR) (Amaral et al., 2019) is also of importance in relation to PD. Systemic and brain IR are found in PD (Aviles-Olmos et al., 2013; Athauda and Foltynie, 2016) and type-2 diabetes (T2D) is a risk factor for the subsequent development of PD (De Pablo-Fernandez et al., 2018). The possible association between PD and T2D lies in their proteinopathic characteristics. Lewy bodies and neurites, composed of amyloid aggregates of misfolded α-synuclein, are the pathological signs of PD (Horvath and Wittung-Stafshede, 2016). T2D also shows pancreatic amyloid deposits, due to the aggregation of islet amyloid polypeptide (amylin). Deposit of amylin accelerates the formation of α-synuclein amyloid (Horvath and Wittung-Stafshede, 2016). Moreover, studies in pancreatic ß-cells of patients with a neuropathological diagnosis of synucleinopathy also supported a direct interaction between amylin and *α*-synuclein (Martinez-Valbuena et al., 2018).

## Studies on the Activity of Melatonin in Animal Models of Parkinson’s disease

Besides the degeneration of DA-containing neurons in the substantia nigra pars compacta (SNpc), PD is considered a progressive disease that affects various neurotransmitter systems. A major argument in this respect is the demonstration that Lewy bodies are located not only in DA neurons but also in raphe nuclei serotonergic neurons, in brainstem noradrenergic neurons and in specific cholinergic neurons all over the CNS (Nutt and Bohnen, 2018). Non-motor symptoms in PD, like gastrointestinal, genitourinary and cardiorespiratory disorders, neuropsychiatric, visual, and sleep-related disorders and anosmia can thus be explained. In fact, since the preclinical non-motor phase of PD can span more than 20 years, the importance of neuroprotection in this regard is evident.

Microglial activation, astrogliosis, and lymphocytic infiltration are the inflammatory patent of PD (Michel et al., 2016). The aggregation of fibrillar α-synuclein is typical of PD and other Lewy body diseases (Marmion and Kordower, 2018). Mitochondrial dysfunction plays a role in this process, as free radicals promote protein folding and aggregation.

Popular animal models of altered brain DA function are the injection of 6-OHDA, or of the neurotoxin MPTP in the nigrostriatal pathway of the rat (Barker and Björklund, 2020). MPTP toxicity is selective for SNpc neurons in non-human primates. Due to its potential to cause disease in humans and in subhuman primates, MPTP is generally preferred to mimic parkinsonism in animals. However, a major drawback of this model is the absence of other neuronal losses in addition to the damaged nigrostriatal dopaminergic system (Marmion and Kordower, 2018).

The effect of melatonin in animal models of PD is summarized in Table 1. From the melatonin doses used in each case, the human equivalent dose of melatonin for a 75°kg adult was calculated by allometry. Allometry is applied to propertie.s whose proportions change as a function of size, in contrast to isometry, whose relation to size is constant (Huang and Riviere, 2014). Body surface area, rather than body weight, is correlated in various mammalian species with numerous biological parameters, including basal metabolism, caloric expenditure, oxygen utilization, blood volume, kidney function and circulating plasma proteins (Reagan-Shaw et al., 2008). Allometry is commonly used to determine doses in phase I human drug clinical trials or to predict drug pharmacokinetic parameters in children, thus decreasing the incidence of toxicity accidents. As summarized in Table 1, theoretical human equivalent doses of melatonin calculated from the results in animals are two–three orders higher than those commonly used in humans.

**TABLE 1:** Effects of melatonin in animal models of PD.

References	Parkinsonism experimental model	Melatonin (dose and administration route	Effects of melatonin	Melatonin equivalent dose for a 75 kg adult patient^a^
Burton et al. (1991)	6-OHDA SNc injections (Wistar rats)	1 and 10 mg/ kg, i.p	Reduced apomorphine-induced rotational behavior	12 and 120 mg
Acuña-Castroviejo et al. (1996)	MPP^+^ injection (C57BL/6 mice)	10 mg/ kg, i.p	Reduced lipid peroxidation and TH-positive neuronal loss in striatum after MPP^+^	60 mg
Jin et al. (1998)	MPP^+^ SNc injection (Sprague-Dawley rats)	10 mg/ kg, i.p	Reduced lipid peroxidation and protected against DA neuronal loss induced by MPP^+^	120 mg
Joo et al. (1998)	6-OHDA striatal injections (Sprague-Dawley rats)	3 and 10 mg/ kg, i.p	Increased striatal DA synthesis and levels	36 and 120 mg
Kim et al. (1998)	6-OHDA striatal injections (Sprague-Dawley rats)	3 or 10 mg/ kg, i.p	Reduced motor deficit and improved dopaminergic neurons survival	36 and 120 mg
DABBENI-SALA et al. (2001)	6-OHDA nigral injections (Sprague-Dawley rats)	50 ± 7.5 μg/ h, s.c	Prevented apomorphine-induced rotational behavior and mitochondrial damage	15 mg
Aguiar et al. (2002)	6-OHDA SNc injections (Wistar rats)	2, 5, 10, and 25 mg/ kg, i.p	Prevented apomorphine-induced rotational behavior and depletion of striatal DA and serotonin levels	24–300 mg
Chen et al. (2002)	MPP^+^ SNc injections (Wistar rats)	10 mg/ kg, i.p.)	Decreased MPP^+^-induced toxicity and recovered GSH levels	120 mg
Khaldy et al. (2003)	MPP^+^ injection (C57BL/6 mice)	5 or 10 mg/ kg i.p	Increase in mitochondrial complex I activity in nigrostriatal neurons	30 and 60 mg
Sharma et al. (2006)	6-OHDA striatal injections (Sprague-Dawley rats)	4 μg/ ml, p.o	Normalized motor deficits and augmented TH immunoreactivity	6 mg
Singh et al. (2006)	6-OHDA striatal injections (Sprague-Dawley rats)	0.5 mg/ kg, i.p	Prevented apomorphine-induced rotational behavior	6 mg
Saravanan et al. (2007)	Rotenone nigral injection (Sprague-Dawley rats)	10, 20, or 30 mg/ kg, i.p	Reduced levels of hydroxyl radicals in mitochondria and increased GSH levels and antioxidant enzymes activities in SNc	120, 240 and 360 mg
Huang et al. (2009)	MPP^+^ SNc injections (Wistar rats)	10 mg/ kg, i.p	Reduced DA neurons apoptosis	120 mg
Tapias et al. (2009)	MPTP injection (C57BL/6 mice)	20 mg/ kg, s.c	Reduced mitochondrial NO levels, reduced lipid peroxidation and improved complex I activity in striatum and SNc	120 mg
Patki and Lau, (2011)	MPTP injections for 5 weeks (C57BL/6 mice)	5 mg/ kg, i.p	Reduced DA neurons loss and locomotor activity deficits. Improved mitochondrial respiration, ATP production, and antioxidant enzyme levels in SNc	30 mg
Singhal et al. (2011)	Maneb plus paraquat (swiss mice)	30 mg/ kg/day, i.p	Reduced lipid peroxidation, TH-positive neurons death, and apoptosis	180 mg
Gutierrez-Valdez et al. (2012)	6-OHDA media forebrain bundle injections (Wistar rats)	10 mg/ kg, p.o	Improved motor performance without causing dyskinesia. Improved DA neurons survival	120 mg
Brito-Armas et al. (2013)	Lentiviral vectors encoding mutant human *a*-synuclein injections in the SNc (Sprague-Dawley)	10 mg/ kg/day, i.p	Improved DA neurons survival	120 mg
Zaitone et al. (2013)	MPTP injections (swiss mice)	5 or 10 mg/ kg/day, p.o	Improved motor performance, striatal DA level, GSH, and antioxidant enzyme activities, and reduced lipid peroxidation. Improved motor response to l-DOPA	30 and 60 mg
Bassani et al. (2014)	Wistar rats were i.p. injected with rotenone	10 mg/kg, i.p	Improved DA neurons survival and increased DA levels	120 mg
Yildirim et al. (2014)	6-OHDA injections into the medial forebrain bundle (Wistar rats)	10 mg/ kg, i.p	Reduced oxidative damage and apoptosis of DA neurons	120 mg
Naskar et al. (2015)	MPTP treatment in BALB/c mice	10, 20 or 30 mg/ kg, i.p	Improved DA neurons survival and enhanced the therapeutic effect of l-DOPA	60, 120 and 180 mg
Ozsoy et al. (2015)	6-OHDA injections into the medial forebrain bundle (Wistar rats)	10 mg/ kg/day, i.p	Improved DA neurons against antioxidant enzyme activities and reduced lipid peroxidation	120 mg
Carriere et al. (2016)	Rotenone injections (Sprague Dawley rats)	4.0 μg/ ml, p.o	Reduced motor deficit and DA neurons loss	6 mg
Li et al. (2017)	6-OHDA nigral injections (Wistar rats)	5 mg/ kg, i.p	Reduced DA neuronal damage	60 mg
López et al. (2017)	C57BL/6 mice receiving MPTP	10 mg/ kg s.c	Preserved mitochondrial oxygen consumption, increased NOS activity and reduced locomotor activity	60 mg
Paul et al. (2018)	Homocysteine SNc injections (Wistar rats)	10, 20 or 30 mg/ kg/day, i.p	Reduced DA loss and improved mitochondrial complex-I activity in SN	120, 240 and 360 mg
Rasheed et al. (2018)	Rotenone injections (Wistar rats)	20 mg/ kg, i.p	Improved motor function by upregulation of tyrosine hydroxylase in striatum. Reduced DA neuron damage	240 mg

^a^ as calculated by normalization of body surface area (Reagan-Shaw et al., 2008) DA = dopamine; 6-OHDA = 6-hydroxydopamine; MPTP = 1-methyl-4-phenyl-1,2,3,6-tetrahydropyridine; MPP+ = 1-methyl-4-phenylpyridinium; i.p. = intraperitoneal; s.c. = subcutaneous; p.o. = oral administration (in drinking water).

In most studies of Table 1 melatonin was administered previously to the toxin and are thus indicating the neuroprotective activity of melatonin to prevent the death of dopaminergic neurons. However, in some studies this neuroprotective effect of melatonin is observed in animal models of PD regardless of the motor benefits. For example, post-treatment with melatonin for 28 days preserved DA levels and tyrosine hydroxylase-positive neurons in rotenone-injured rats and improved depressive behavior in the absence of significant improvement in motor deficit (Bassani et al., 2014). Furthermore, when evaluating the effects of a slow-release melatonin preparation by intracerebroventricular implants in rats injected with 6-OHDA or MPTP, melatonin actually impaired motor performance (Willis and Armstrong, 1999). These effects could be explained by the inhibition of DA release given by high concentrations of the methoxyindole (Zisapel et al., 1982). Regardless of the discrepancies cited, the preventive activity of melatonin in neurodegeneration related to PD is generally accepted (Lin et al., 2007; Chang et al., 2012; Leeboonngam et al., 2018).

The accumulation and spread of oligomeric forms of neurotoxic α-synuclein could be explained by an insufficient autophagic-lysosomal clearance (Boland et al., 2018). In addition, other pathways of clearance are disrupted, such as the ubiquitin-proteasome system, chaperone-mediated autophagy, extracellular protease clearance, or the glymphatic system (Boland et al., 2018). The expression of the water channel aquaporin 4 (AQ4) in the glymphatic system is severely altered in PD brains (Hoshi et al., 2017) and this may explain why the levels of α-synuclein in the CSF are inversely correlated with symptoms in PD patients (Schirinzi et al., 2019). The association of sleep loss with impaired glymphatic clearance is important in PD because RBD is a prodrome of PD. In mice, injection of melatonin to animals increased glymphatic clearance (Pappolla et al., 2018). In PD patients melatonin preserves sleep (Daneshvar Kakhaki et al., 2020).

DA supplementation by the administration of its precursor l-DOPA is an effective treatment to reduce motor symptoms in PD patients. However, long-term administration of -l-DOPA causes undesired motor sequels like dyskinesias (Remington, 2007). Furthermore, the production of neurotoxins like 6-OHDA is detected after administration of a high dose of l-DOPA. Melatonin, but not l-DOPA, restored the neuronal density in the striatal column of MPTP-treated mice strongly supporting the use of melatonin as an adjunct to l-DOPA therapy in PD patients (Naskar et al., 2015). In the MPTP monkey model of PD, the combined treatment of melatonin and l-DOPA significantly reduced nighttime sleep fragmentation and daytime sleep episodes, indicating that melatonin treatment can be useful in treating sleep disorder in patients with PD (Belaid et al., 2015).

## Clinical Use of Melatonin in Parkinson’s disease and RBD

A phase advance in nocturnal melatonin secretion was observed in PD patients treated with l-DOPA (Fertl et al., 1993; Bordet et al., 2003). Patients treated with l-DOPA showed increased melatonin secretion during the day, perhaps as an adaptive response to neurodegeneration (Bordet et al., 2003). PD patients, particularly those showing excessive daytime sleepiness, exhibited a reduced amplitude of the circulating melatonin rhythm (Videnovic et al., 2014). A chronobiologic therapy for non-motor manifestations of PD, comprising the scheduled exposure to bright light in the morning and melatonin administration at sleep time, can thus be recommended.

The postulation of an association between motor fluctuations in PD and the diurnal variation in circulating melatonin levels relies on the existence of melatonin effects on striatal DA and serotonin content (Escames et al., 1996). In view that l-DOPA-related motor complications is seen in about 50% of PD patients after 5°years of treatment, the results obtained in experimental parkinsonism warrant the use of melatonin to decrease l-DOPA doses in PD (Naskar et al., 2015).

In view that the stimulation of the globus pallidus ameliorated motor symptoms and complications in PD and inhibited the increase in diurnal plasma levels of melatonin the hypothesis was put forth that wearing-off episodes in PD could be related to the loss of the inhibitory motor effect of melatonin (Catalá et al., 1997). Relevant to the topic of the present review, epidemiological studies indicated that longer years of night work were associated with a reduced risk of PD and decreased levels of circulating melatonin (Bajaj et al., 2010).

PD patients showed a decreased density of MT1 and MT2 melatonin receptors in the substantia nigra and amygdala (Adi et al., 2010b) a finding that led to the assumption that an altered melatonergic system could underlie the altered sleep/wake cycle observed in PD. An increase in nighttime sleep was detected actigraphically in 40 PD patients receiving a daily dose of 50 mg melatonin at bedtime while those patients treated with 5 mg of melatonin daily reported a significant improvement in subjective sleep assessment only (Dowling et al., 2005).

Medeiros and co-workers examined 18 parkinsonian patients who were randomized after a baseline polysomnography to receive melatonin (3 mg) or placebo at bedtime for 4°weeks (Medeiros et al., 2007). A significant improvement of sleep quality but no effect on motor dysfunction was found. A similar dissociation of effects of melatonin was reported in a double-blind, placebo, randomized study in 34 PD patients (Ahn et al., 2020). The beneficial effects on sleep quality after melatonin were associated with improved non-motor symptoms and quality of life but not with amelioration of motor symptoms.

However, in another double-blind, placebo, randomized study, 10 mg of melatonin to 60 PD patients for 12°weeks significantly normalized Unified Parkinson’s disease Rating Scale part I score, PSQI, Beck Depression and Anxiety Inventories, and plasma levels of C-reactive protein, total antioxidant capacity, glutathione, LDL-cholesterol, glucose and insulin (Daneshvar Kakhaki et al., 2020). Gene expression of TNF-α, PPAR-γ and LDLR was also corrected by melatonin. In partial agreement with this last study, UPDRS score decreased as well as COX-2 activity, nitrates/nitrites and lipoperoxides in melatonin-treated PD patients (25 mg every 12 h) (Ortiz et al., 2017).

A reduction of stiffness and bradykinesia, agitation and psychiatric side effects were reported in parkinsonian patients exposed to 1–1.5 h of light (1000–1500 lux) before bedtime, the authors concluding that decreased melatonin levels could be of therapeutic value in PD (Willis and Turner, 2007). However, as shown in depressive patients undergoing phototherapy, the inhibition of melatonin release is not the likely mechanism by which artificial light exerts its therapeutic effect (Lewy et al., 1984). Collectively, the gathered information points out to the circadian apparatus as an important diagnostic and therapeutic tool in PD (Willis and Freelance, 2018). A summary of clinical trials with melatonin in PD is offered in Table 2.

**TABLE 2:** Clinical trials with melatonin in Parkinson’s disease.

Ref	Sample	Design	Comparator	Duration	Melatonin treatment	Assessments	Main results
Dowling et al. (2005)	40 PD	Open-label	Placebo	2 weeks	5–50 mg/day at bedtime, p.o	Actigraphy	Melatonin 50 mg significantly increased nighttime sleep as compared to placebo. Melatonin 5 mg improvement significantly subjective sleep quality as compared to placebo or melatonin 5 mg
Medeiros et al. (2007)	18 PD	Open-label	Placebo	4 weeks	3 mg/day at bedtime, p.o	PSG, PSQI, and ESS	Melatonin increased sleep latency by 50%, REM sleep without atonia by 66%, and reduced sleep efficiency by 72%. Subjective quality of sleep was also improved
Litvinenko et al. (2012)	38 PD (no dementia, self-reported sleep disorders)	Open-label	Clonazepam 2 mg	6 weeks	3 mg/day at bedtime, p.o	PSG, PDSS, Neuropsychological testing (MMSE, five-word test, digit span and the Hamilton scale)	Melatonin had a greater effect on sleep disorders compared to clonazepam. Patients treated with melatonin had better scores on the MMSE, five-word test, and the Hamilton scale at the end of the study
Ortiz et al. (2017)	13 PD	Double-blind, randomized	Placebo	12 months	25 mg every 12 h, p.o	UPDRS. Measurement of. COX-2 activity and markers of antioxidants activity	Melatonin decreased COX-2 activity and improved some antioxidant markers. UPDRS score decreased in the melatonin-treated patients but no in the placebo group
Daneshvar Kakhaki et al. (2020)	60 PD	Double-blind, randomized	Placebo	12 weeks	10 mg/day at bedtime, p.o	UPDRS, PSQI, BDI, Bai. Markers of antioxidant activity, inflammatory response, and hormonal status were also measured	Melatonin supplementation significantly reduced UPDRS part I score, PSQI, BDI and Bai. It also resulted in an increase in antioxidant capacity, and reduced serum insulin levels, HOMA-IR, total and LDL-cholesterol as well as gene expression of TNF-α, PPAR-γ and LDLR.
Ahn et al. (2020)	34 PD (poor sleep quality)	Double-blind, randomized	Placebo	4 weeks	2 mg prolonged release melatonin (Circadin^R^) at bedtime	PSQI, RBDSQ,, the ESS, NMSS, PDQ-39, and UPDRS-III	Melatonin treatment was associated with improvements in PSQI, NMSS and PDQ-39. No changes were observed in UPDRS-III.
Delgado-Lara et al. (2020)	26 PD	Double-blind, randomized	Placebo	3 months	25 mg/day at bedtime, p.o	ESS, SCOPA-sleep, UPDRS and Hoehn and Yahr scale. Relative expression of the PER1 and BMAL1 genes (RT-qPCR)	Melatonin increasing BMAL1 expression but did not improved sleep parameters

p.o.: oral administration; PSG = polysomnography; PSQI = pittsburgh sleep quality index; ESS = epworth sleepiness scale; PDSS = PD sleep scale; UPDRS = unified parkinson’s disease rating Scale; COX-2 = ciclooxigenase two; BDI = beck depression Inventory; BAI = beck anxiety inventory; HOMA-IR = homeostatic model assessment for insulin resistance; RBDSQ = rapid eye movement sleep behavior disorder screening questionnaire; NMSS = non-motor symptoms scale; PDQ-39 = Parkinson’s disease quality of life-39.

Table 3 summarizes the clinical studies reporting on the use of melatonin in RBD. Non-motor symptoms such as RBD, hyposmia or depression precede the onset of PD by several years (Michel et al., 2016). RBD prevalence in PD amounts to 20–50% while in the general population is about 1%. Patients with RBD, especially men >50 years of age, will convert to an α-synucleinopathy, with a mean interval for conversion from time of RBD onset of approximately 10°years (Dauvilliers et al., 2018). As above discussed, sleep/wake cycle disturbances are common non-motor symptoms in PD and presumably reflect the more fundamental circadian pathology in parkinsonian patients.

**TABLE 3:** Studies including RBD patients with melatonin.

Ref	Nb of RBD patients	Design	Study´s duration	Melatonin treatments	Measured	Results
Kunz and Bes, (1997)	1	Case report	5 months	3 mg/day at bedtime, p.o	Actigraphy, PSG	Significant reduction of motor activity during sleep, as measured by actigraphy. PSG showed an increase of REM sleep
Kunz and Bes, (1999)	6	Case series	6 weeks	3 mg/day at bedtime, p.o	PSG	Significant PSG improvement in 5 patients
Takeuchi et al. (2001)	14	Case series	Variable	3–9 mg/day at bedtime, p.o	PSG	13 patients reported a no problematic sleep behaviors after melatonin administration. A decrease in tonic REM activity after melatonin administration was also observed
Boeve et al. (2003)	14	Case series (retrospective)	14 months	3–12 mg/day at bedtime, p.o	PSG	8 patients experienced continued benefit
Anderson and Shneerson, (2009)	39	Case series (retrospective)		10 mg/day at bedtime p.o	Medication use	Two patients used melatonin (10 mg) and both found it effective
McCarter et al. (2013)	25	Case series (retrospective)	27–53 months	6 mg/day at bedtime, p.o		Melatonin users reported reduced injuries and fewer adverse effects
Kunz and Mahlberg, (2010)	8	Double blind, randomized, placebo-controlled	4 weeks	3 mg/day at bedtime, p.o	PSG	Reduced REM without atonia and frequency of RBD episodes
Kunz and Bes, (2018)	1	Case report	5 years	2 mg/day at bedtime, p.o. (prolonged release formulation)	PSG and DA transporter scintigraphy (DaTSCAN)	A 72-year-old man was clinically suspected to suffer from PD in 2011. PD and RBD diagnoses were confirmed by DaTSCAN and PSG. 6-months after melatonin treatment, clinical signs of RBD were absent. Control PSG in 2014 confirmed normalized REM sleep with atonia. DaTSCANs performed in 2013 and 2015 indicated normalization of DA transporter density
Schaefer et al. (2017)	4 (with concomitant obstructive sleep apnea)	Open label	4 weeks	2 mg/day at bedtime, p.o. (prolonged release formulation)	PSG	Treatment led to a relevant clinical improvement of RBD symptoms in all patients
Gilat et al. (2020)	30	Double blind, randomized, placebo-controlled	8 weeks	4 mg prolonged release melatonin p.o./daily at bed time	Aggregate of RBD incidents averaged over weeks 5–8 of treatment captured by a weekly diary	No differences between groups at the primary endpoint

RBD= REM sleep behaviour disorder; PSG= polysomnography

As summarized in Table 3 the daily doses of melatonin effective to treat RBD ranged from 3 to 12 mg daily at bedtime. A decreased in movement time during REM sleep and in number of R epochs without atony were documented by polysomnography in RBD patients treated with melatonin; in contrast patients receiving clonazepam exhibited the persistence of muscle tone in REM sleep. Based on these results, a clinical consensus recommended the use of melatonin in RBD at level B (Aurora et al., 2010). However, a negative result was recently published in a randomized, double-blind, placebo controlled, parallel-group trial of 30 RBD patients treated unsuccessfully for 8°weeks with 4 mg prolonged release melatonin (Gilat et al., 2020). Therefore, more high-quality studies are needed in this respect.

In view of the high conversion rate of idiopathic RBD to PD several consensuses have stressed the necessity of studies looking for neuroprotection in RBD. In fact, the rate of conversion to synucleinopathy in RBD patients treated with clonazepam is very high (Dauvilliers et al., 2018). Although comparable data are not yet available for RBD patients treated with melatonin, a case report deserves consideration (Kunz and Bes, 2018). In a 72-year-old male RBD patient treated with 2 mg slow-release melatonin daily an increase in DA transporter density over successive years was documented. Moreover, the clinical and electrophysiologic signs of RBD disappeared after 6°months. In addition, no signs of PD were present 4°years after the first examination, indicating the possible neuroprotective role for melatonin in synucleinopathy (Kunz and Bes, 2018).

**FIGURE 1:**
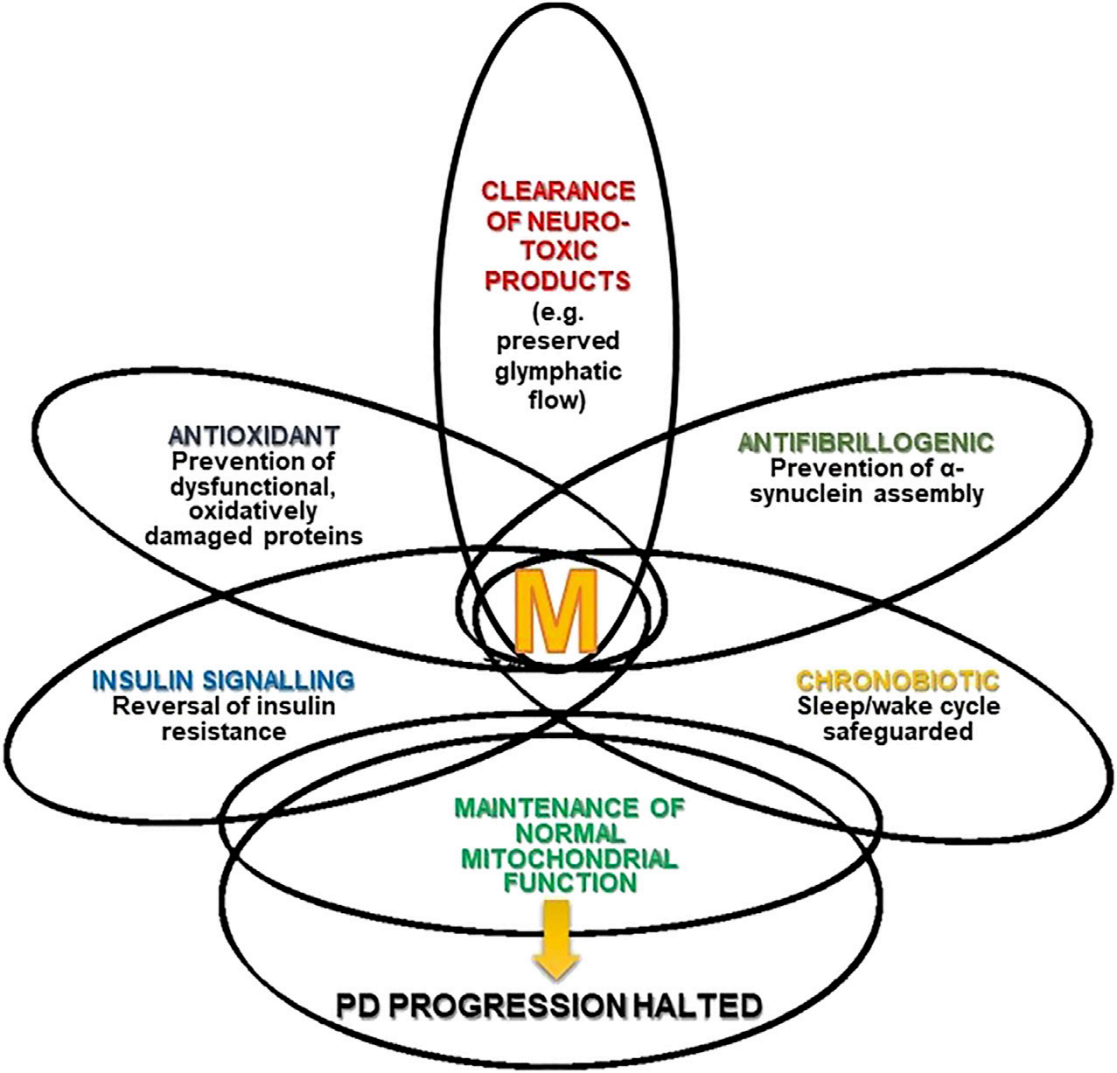
Melatonin (M) and Parkinson’s disease (PD). The Figure depicts the multiple effects of melatonin and the different degree of overlap (interrelations and mutual influences) they have.

Figure 1 summarizes the different mechanisms by which melatonin can stop the progression of PD. The intersections in the Figure represent the multiple effects of melatonin and the different degrees of overlap (interrelationships and mutual influences) discussed in this review article.

## Conclusion

Due to the hypnotic and chronobiotic properties of melatonin, its use for the treatment of insomnia has been recommended. Several meta-analyses support such a therapeutic role (Auld et al., 2017; Ferracioli-Oda et al., 2018; Li et al., 2019) Additionally, a number of consensuses concluded that melatonin is the first-line treatment when a hypnotic is indicated in patients over 55 years of age (Wilson et al., 2010; Geoffroy et al., 2019; Palagini et al., 2020; Vecchierini et al., 2020). However, as discussed in this article, clinical studies with 2–5 mg melatonin/day may not be adequate to provide comparison with data on protection against neurodegeneration derived from animal studies. Indeed, studies with doses of 100 mg/day or higher are needed. Melatonin may also be involved in the pathophysiology of other non-motor symptoms in PD, but the current evidence is not convincing enough (Li et al., 2020; Batla et al., 2021) Therefore, more research is needed.

The safety of melatonin is very high and its non-toxicity remarkable. The lethal dose 50 after intraperitoneal injection was 1168 mg/kg (rats) and 1131 mg/kg (mice) but could not be reached after oral administration of melatonin (tested up to 3200 mg/kg in rats or subcutaneous injection of melatonin (tested up to 1600 mg/kg in rats and mice) (Sugden, 1983). There is evidence in dose escalation, phase 1, experiments of the remarkable lack of toxicity of melatonin in humans up to 100 mg (Galley et al., 2014; Andersen et al., 2016). As discussed elsewhere (Cardinali, 2019a), high doses of melatonin have been used in various pathologies without undesirable sequelae, that is, in humans, melatonin has a high safety profile and, in general, is very well tolerated. Currently, the only option for the incumbent physician interested in the use of melatonin as a cytoprotector is the off-label indication of the drug. Therefore, studies on the potential disease-modifying effects of melatonin are warranted. RBD patients may be the first group to target with melatonin. Indeed, melatonin has shown some interesting therapeutic effects in this group, although it must be remarked that high-quality evidence is lacking. Therefore, two birds might be killed with the same tone, as melatonin would offer an immediate clinical benefit in addition to reducing the rate of conversion to PD.
